# Optical fibre based real-time measurements during an LDR prostate brachytherapy implant simulation: using a 3D printed anthropomorphic phantom

**DOI:** 10.1038/s41598-021-90880-6

**Published:** 2021-05-27

**Authors:** P. Woulfe, F. J. Sullivan, L. Byrne, A. J. Doyle, W. Kam, M. Martyn, S. O’Keeffe

**Affiliations:** 1 Optical Fibre Sensors Research Centre, University of Limerick, Limerick, Ireland; 2 Health Research Institute, University of Limerick, Limerick, V94 T9PX Ireland; 3 Department of Radiotherapy Physics, Galway Clinic, Galway, Ireland; 4 Prostate Cancer Institute, Galway Clinic, Galway, Ireland; 5 Department of Radiotherapy, Galway Clinic, Galway, Ireland; 6 School of Physics, FOCAS, Technological University Dublin, Dublin, Ireland

**Keywords:** Optics and photonics, Physics

## Abstract

An optical fibre sensor based on radioluminescence, using the scintillation material terbium doped gadolinium oxysulphide (Gd_2_O_2_S:Tb) is evaluated, using a 3D printed anthropomorphic phantom for applications in low dose-rate (LDR) prostate brachytherapy. The scintillation material is embedded in a 700 µm diameter cavity within a 1 mm plastic optical fibre that is fixed within a brachytherapy needle. The high spatial resolution dosimeter is used to measure the dose contribution from Iodine-125 (I-125) seeds. Initially, the effects of sterilisation on the sensors (1) repeatability, (2) response as a function of angle, and (3) response as a function of distance, are evaluated in a custom polymethyl methacrylate phantom. Results obtained in this study demonstrate that the output response of the sensor, pre- and post-sterilisation are within the acceptable measurement uncertainty ranging from a maximum standard deviation of 4.7% pre and 5.5% post respectively, indicating that the low temperature sterilisation process does not damage the sensor or reduce performance. Subsequently, an LDR brachytherapy plan reconstructed using the VariSeed treatment planning system, in an anthropomorphic 3D printed training phantom, was used to assess the suitability of the sensor for applications in LDR brachytherapy. This phantom was printed based on patient anatomy, with the volume and dimensions of the prostate designed to represent that of the patient. I-125 brachytherapy seeds, with an average activity of 0.410 mCi, were implanted into the prostate phantom under trans-rectal ultrasound guidance; following the same techniques as employed in clinical practice by an experienced radiation oncologist. This work has demonstrated that this sensor is capable of accurately identifying when radioactive I-125 sources are introduced into the prostate via a brachytherapy needle.

## Introduction

A common treatment option for prostate cancer is low dose rate (LDR) seed brachytherapy, which has been shown to have excellent long-term outcomes^1^. The main advantage of the technique is its use of a higher dose of radiation in a more targeted area, compared with external beam radiotherapy^2^. A real-time intraoperative guided trans-perineal LDR prostate brachytherapy technique, popularized by Stone and Stock^3^, is employed in this work. Good technique is required to ensure optimal dosimetry, and acceptable short as well as long term outcomes^3^. Trans-rectal ultrasound (TRUS) guidance is utilised during implantation to visualise the prostate and surrounding anatomy, and to guide the insertion of needles, through which brachytherapy seeds are delivered. TRUS imaging provides excellent soft tissue visualisation, making it ideal for applications in the treatment of prostate cancer. However, due to the limited spatial resolution of ultrasound (US) transceivers, and due to the low echogenic nature of metallic seeds, the identification of seed locations is often difficult^4^. Accurate knowledge of implanted seed location is crucial when assessing adherence to the employed dosimetric criteria; ensuring adequate dose to the prostate (D_90_, V_100_, V_150_), while also minimising dose to the organs at risk (D_30_ urethra, as well as D_2cc_ rectum), in line with international guidelines^5^.

The aim of this study is to perform in vitro measurements using an optical fibre based sensor, in a 3D printed anthropomorphic phantom. This work therefore acts as a proof of concept for ultimately employing an optical fibre based system for real time in vivo dosimetry (RTIVD); enabling treatment interruption if measured doses [derived from the photon counting rate (PCR)] differ significantly from the treatment plan. An optical fibre based system employed in this way could be used as a radiation protection tool and as a treatment quality assurance (QA) tool. Measurements are limited only by the accuracy of the dosimeter and the knowledge of its position within the patient^6^.

## Materials

### I-125 source

The seeds used in LDR brachytherapy, within our clinical setting, are typically Iodine-125 (I-125), with a half-life of 59.43 days. Once again, within our clinical setting, typical apparent activities employed range from 0.357 to 0.42 mCi, and the typical number of seeds employed range from 60 to 80 seeds, depending on the volume of the prostate. Theragenics Co., I-Seed I-125, AgX100 were used in the present study. Source dimensions for the AgX100 seeds are detailed in Mourtada et al^7^. The mean photon energy on the surface of an AgX100 seed has been calculated as 27.29 keV in the Carleton Laboratory for Radiotherapy Physics (CLRP) TG-43 parameter database, with statistical uncertainties < 0.01%^8^.

### Terbium doped gadolinium oxysulphide optical fibre dosimeter

**Figure 1:**
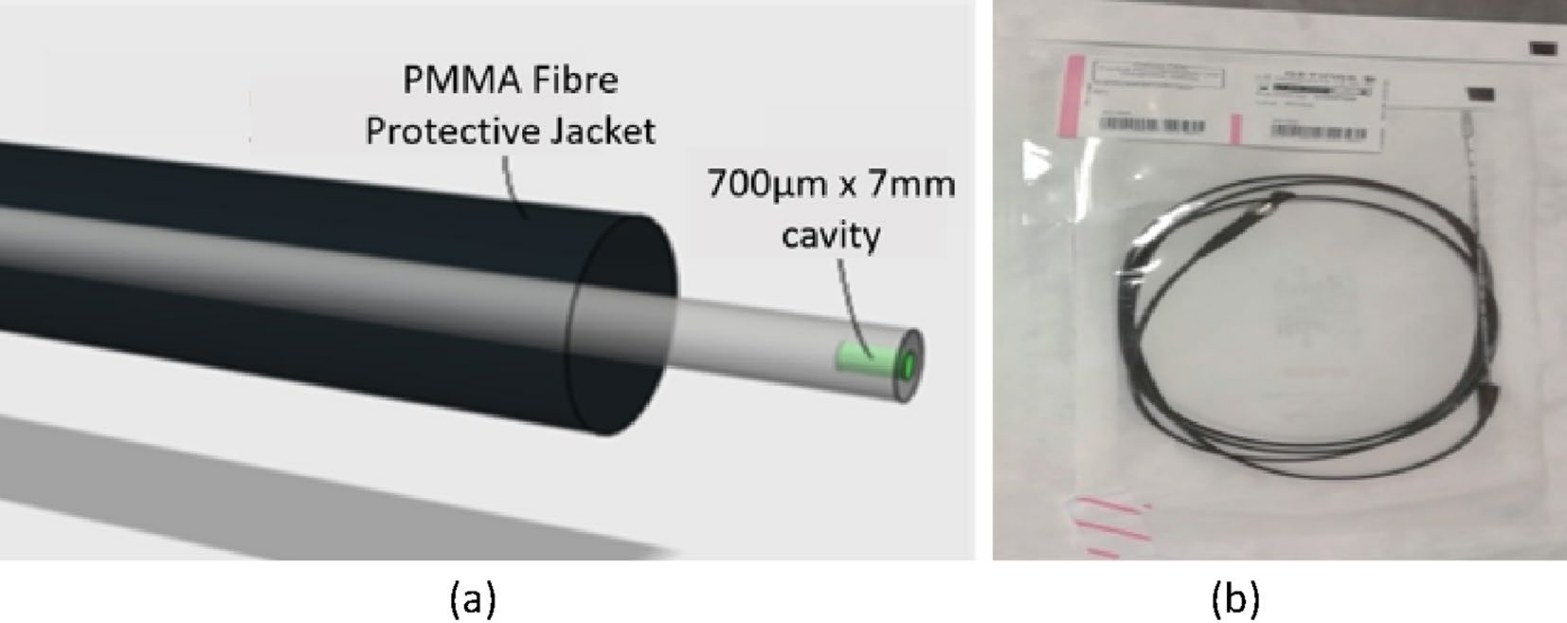
Optical fibre based radiation dosimeter: (**a**) schematic of sensor design, (**b**) optical fibre sensor within brachytherapy needle in the sterilized packages.

The optical fibre sensor, shown in Fig. 1a, is constructed by micromachining a cavity in the 1 mm core of a polymethyl methacrylate (PMMA) plastic optical fibre. The cavity, 700 μm in diameter and 7 mm in depth, is filled with a scintillating material, terbium doped gadolinium oxysulphide (Gd_2_O_2_S:Tb, GOS) and sealed with a Henkel Loctite Hysol M-31CL Medical Device Epoxy. The scintillation material fluoresces on exposure to ionising radiation and the resultant emitted fluorescent light penetrates the PMMA optical fibre core and propagates along the fibre to a Hamamatsu Multi-Pixel Photon Counting Module (MPPC) C13366^9^ for monitoring of the optical signal. The data was captured using proprietary software of the MPPC with a gate time setting of 100 ms and 0.5 photo-electron threshold. The gate time represents the time duration within which the photon counts are integrated, within the MPPC module. The data presented in this work is the optical signal captured in the presence of an I-125 seed, minus the dark count rate (DCR) captured at near zero light input. The small dimensions of the sensor^10,11^, with an overall outer diameter of 1 mm, allow for it to be guided within existing brachytherapy equipment (e.g. within the brachytherapy needle), as shown in Fig. 1b. This will allow the sensor to be located directly within the prostate, using techniques the radiation oncologist is already familiar with.

### Sterilization

The STERRAD® NX System^12^ developed by Advanced Sterilization Products (ASP), a Johnson & Johnson company, uses both hydrogen peroxide vapor and low-temperature gas plasma to rapidly sterilize the dosimeters. Since the load temperatures do not exceed 55 °C and sterilization occurs in a low moisture environment, the STERRAD® NX System is particularly suited to the sterilization of heat and moisture-sensitive instruments. The process that occurs is as follows: the dosimeter to be sterilized is placed in the sterilization chamber, the chamber is closed, aqueous hydrogen peroxide is delivered to the vaporizer/condenser, and evacuation begins. The overall sterilization process was repeated twice. Figure 1b above depicts the sterilized dosimeters packaged for use.

### Phantoms

Two phantoms were utilised during the course of this evaluation. The first, a custom PMMA (also known as Lucite, Plexiglas or Perspex) phantom was used to evaluate the effects of the sterilisation process on the sensor response. The second, an anthropomorphic 3D printed training phantom, was used to validate the sensor for RTIVD.

#### PMMA Phantom

**Figure 2:**
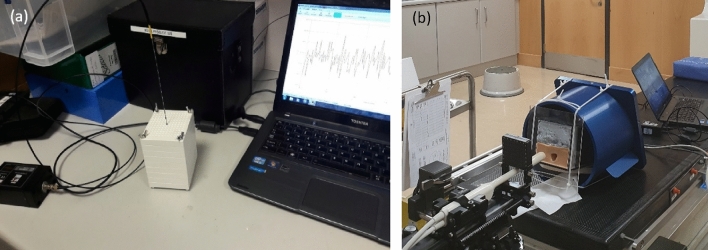
Phantoms used during sensor evaluation: (**a**) PMMA Phantom, (**b**) 3D printed anthropomorphic phantom.

This PMMA Phantom, shown in Fig. 2a, has a density of 1180 kg m^−3^, and was used to evaluate the effect of the sterilisation process on the optical fibre sensor. The phantom design has outer dimensions of 80 × 80 × 90 mm^3^, created from 10 mm thick slabs stacked together, and a central hole to accommodate the dosimeter. An array of 13 × 13 holes, each with a diameter of 1 mm, were machined to replicate a prostate biopsy template used in LDR Brachytherapy. To provide full scatter conditions, the I-125 source was surrounded by a sufficient amount of phantom material^13^. Thus, during irradiation, the PMMA sheet containing the I-125 sources and optical fibre were contained between four further sheets of PMMA, resulting in a depth of 4 cm. A 90 mm thick phantom is considered to offer adequate backscatter for low energy brachytherapy sources as we have seen range of detection limited to 30 mm^14^. During irradiation, the longitudinal axes of both the sensor and the I-125 seed are parallel, with their centres aligned.

#### Anthropomorphic 3D printed training phantom

This paper also utilises an anthropomorphic, 3D printed training phantom for LDR brachytherapy for prostate cancer, described by Doyle et al^15,16^. In contrast to those found commercially, this phantom can be used to plan and validate treatment tailored to an individual patient. The phantom, shown in Fig. 2b was used as a test bed for the optical fibre sensor. The high-fidelity phantom replicated the soft tissue characteristics of the male pelvis and facilitated needle puncture and the introduction of both I-125 seeds and the optical fibre sensor contained within a brachytherapy needle. The sensor is introduced through the brachytherapy needle grid to monitor in real-time the photon count rate (PCR) during the procedure.

## Methods

Testing was performed at the Radiotherapy Department, Galway Clinic, Galway. The first of these experiments was to examine the performance of the dosimeter pre-sterilisation versus post-sterilisation by investigating the following: (1) Repeatability, (2) response as a function of angle, and (3) response as a function of distance to demonstrate sensitivity. This work was carried out to identify any issues relating to the sterilisation process on the sensor performance, prior to the evaluation of the sensor for in vitro measurements using the anthropomorphic phantom.

The optical fibre dosimeter was fixed within the central hole of the PMMA Phantom at a depth of 4 cm as depicted in Fig. 2a. The brachytherapy seeds were inserted into the PMMA Phantom for a fixed period of time and the response of the sensor was monitored. The phantom set-up was designed such that the centres of the radiation source and the optical fibre sensor were aligned. The sensor was initially tested for its response to one 0.410 mCi I-125 seed, for a comparison of optical signal, pre/post-sterilisation. In this particular investigation, the repeatability of the sensor was evaluated by removing and re-introducing the sensor over three consecutive cycles at a distance of 5 mm (± 1 mm positional uncertainty) from the radiation source. The second investigation sought to assess the response of the optical fibre sensor (OFS) as a function of angle with respect to the seed, which was again positioned 5 mm from the sensor. Four angles were evaluated, along the plane perpendicular to the centre of the sensors longitudinal axis; 0° (Top), 90° (Right), 180° (Bottom) and 270° (Left). Finally, we examine the sensitivity of the sensor as a function of distance from a single seed, over the range 5–30 mm, in 5 mm steps. The dose fall-off with distance from the radiation source can be described using the TG-43 formulism^13^, via the VariSeed (Varian Medical Systems) treatment planning system (TPS) [Version 8.0.2], and compared to the photon counting rate (PCR) fall-off measured with the sensor. Each of the investigations described above were performed with the sensor pre-sterilisation and post-sterilisation.

**Figure 3:**
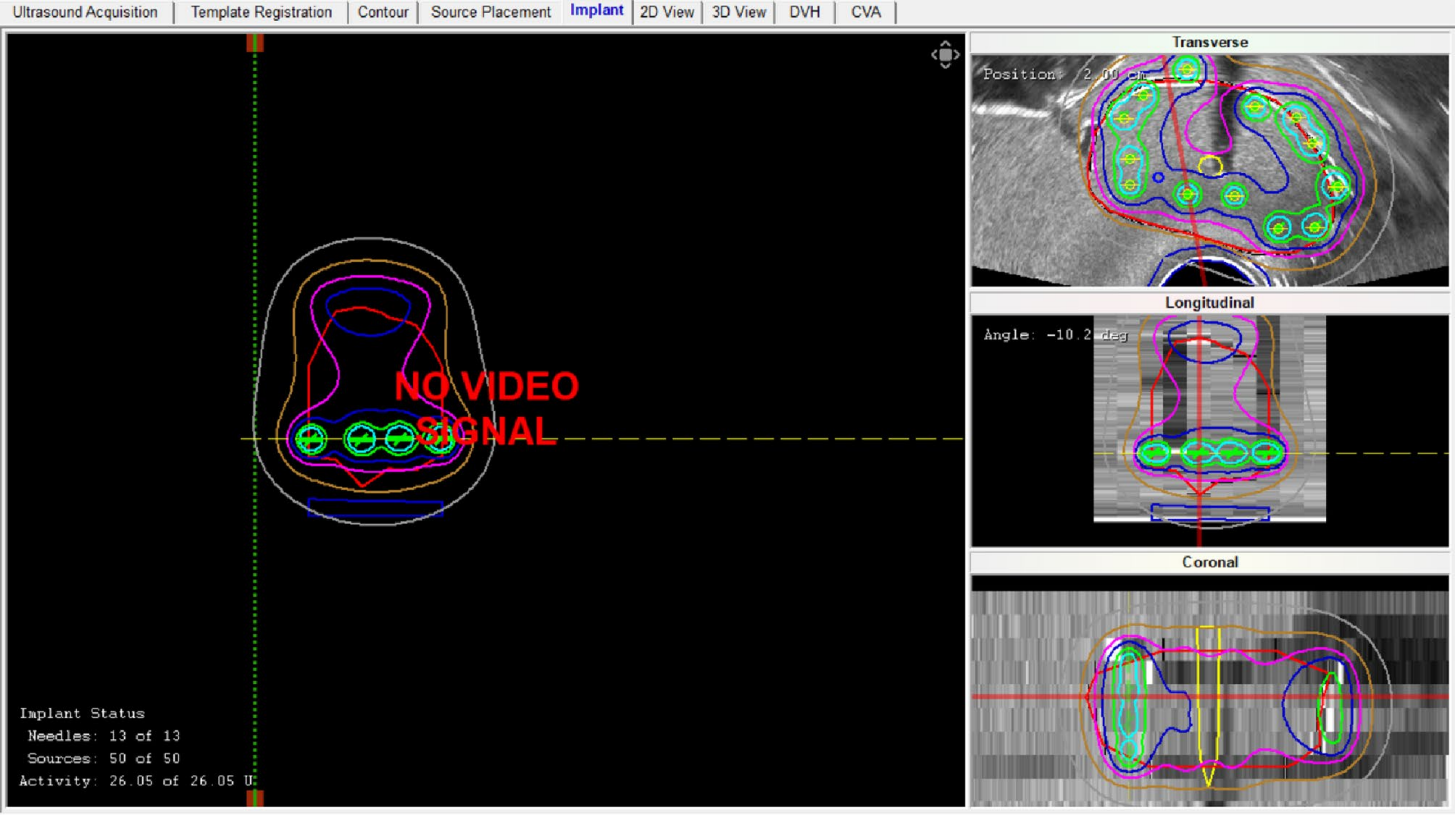
3D anthropomorphic phantom treatment plan in the VariSeed TPS. Post-implant dosimetry analysis displaying transverse, longitudinal, and coronal views (without live video signal): I-125 seeds (green), prostate (red), urethra (yellow), and isodose lines are displayed.

For the second part of the study, a realistic clinical prostate brachytherapy case was simulated using a 3D printed training phantom, this phantom was printed based on patient anatomy with exact volume and dimensions of the prostate to represent the patient. One of the authors (FS), an experienced radiation oncologist, performed the simulation using exactly the same techniques employed in clinical practice^17^. A Hitachi Preirus (Hitachi Medical Solutions) US system was used to acquire the images of the 3D printed training phantom. Steps involved in the simulation process were setting the prostate symmetrically within the grid, identifying the base of the prostate on sagittal imaging, identifying the apex of the prostate on both sagittal and axial imaging, determining the length and volume of the prostate, and acquiring the images required for treatment planning by using a 5 mm stepping device (Civco Inc). Images are captured within the VariSeed TPS via a direct link with the US system. During the implant procedure, 3–4 seeds were evenly spaced throughout the length of the simulated prostate for each needle (see Fig. 3), to achieve the desired dosimetric coverage (50 peripheral seeds were implanted in total). The entire implantation procedure was recorded and a timestamp was assigned to each seed to provide accurate time correlation with the real-time photon counting rate (PCR) from the sensor.

**Figure 4:**
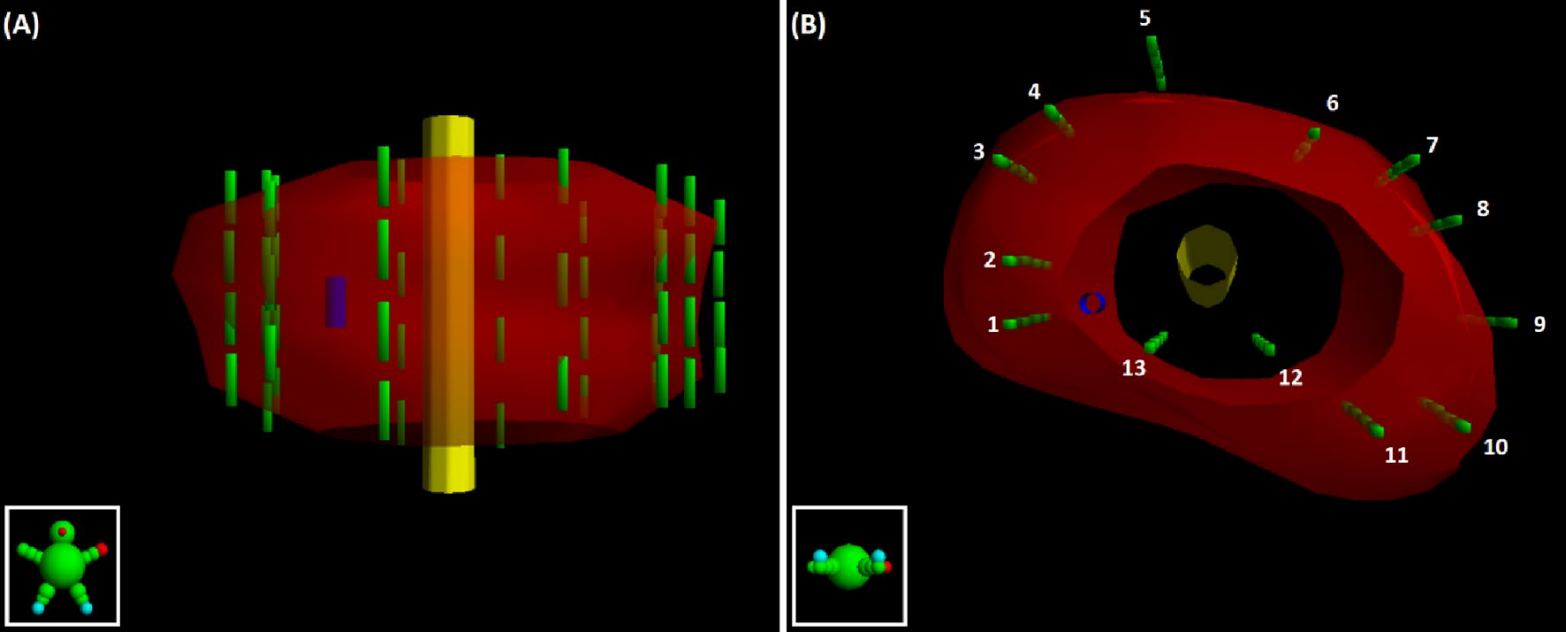
(**a**) Coronal view of structures/sources in VariSeed TPS: sensor (blue cylinder), prostate (red), urethra (yellow), and I-125 seeds (green). (**b**) Transverse view of structures / sources with needle numbers also identified.

Within the VariSeed TPS, shown in Fig. 4, a structure was added at a position corresponding to C2.5 on the template grid to represent the position of the sensor (blue cylinder). This cylindrical sensor structure has a diameter of 2 mm (accounting for the 1 mm outer diameter of the optical fibre and a positional uncertainty of approximately ± 1 mm) and a length of 5 mm (dictated by the 5 mm slice spacing employed during image acquisition). Figure 4 also provides a representation of the position and distribution of the implanted I-125 seeds (green)/needles with respect to the sensor. Using VariSeed the “expected” dose to the sensor structure was calculated for each of the 50 seeds that were inserted into the periphery of the prostate. This allows for a relative comparison of the “accumulated dose” to the sensor structure, within the TPS, and the real-time PCR from the sensor, as a function of time. The data presented in this work represent the PCR minus the dark count rate (background noise). The objective of this work was to identify if the sensor was capable of accurately identifying when radioactive I-125 sources were introduced into the simulated prostate for each peripheral needle (i.e. is there a noticeable increase in the PCR per needle).

## Results

### Sterilisation effects

The average PCR pre- and post-sterilisation was 1290 and 1307 counts per gate (c.p.g.) respectively (above the DCR of approximately 1100 c.p.g); these figures represent the average of the mean PCR for each individual measurement, integrated over 170 s. The pooled standard deviation (SD) of the PCR pre- and post-sterilisation are 59 and 69 c.p.g. respectively; where the pooled SD is defined as the root mean square of the standard deviations for each individual measurement, integrated over 170 s. The response of the sensor as a function of angle with respect to the radiation source are presented in Table 1. PCR measurements acquired at each angle agree within measurement uncertainty (defined by the SD), both pre- and post- sterilisation.

**Table 1:** Mean and SD of the PCR for each angle considered.

Angle	Pre-sterilisation			Post-sterilisation		
	Mean (c.p.g.)	SD (c.p.g.)	SD (%)	Mean (c.p.g.)	SD (c.p.g.)	SD (%)
Top—0°	1323	61	4.6	1368	60	4.4
Bottom—180°	1274	62	4.9	1239	70	5.6
Right—90°	1294	58	4.5	1232	60	4.9
Left—270°	1294	70	5.4	1229	71	5.8

**Figure 5:**
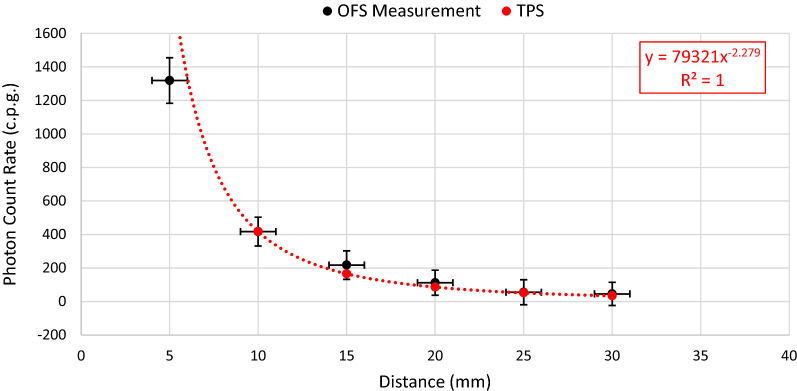
Average PCR measurement data (black circles) as a function of distance from a single I-125 seed obtained pre- and post-sterilisation. Theoretical data generated using the VariSeed TPS (red circles) are normalised to the PCR measurement value at 10 mm. The dashed red line represents a power trendline fit to the theoretical data, with the equation of the line shown in the top right corner of the graph in red text.

PCR fall-off as a function of distance was also considered with the results illustrated in Fig. 5. Based on the findings of the repeatability measurements, measured data in Fig. 5 represent the average of the PCR signal obtained with the sensor both pre and post sterilization, since the sterilization process has been shown to have no significant impact on the measurement signal. PCR data were integrated over a period of 130 s, at each given distance, for both the pre and post sterilization measurements. The vertical error bars on the measurement data represent the pooled SD of the PCR (multiplied by two for 95% confidence), integrated over a period of 130 s, at each distance. The horizontal error bars on the measurement data represent a ± 1 mm positional uncertainty. The expected dose fall-off rate was calculated using the VariSeed TPS, for the AgX100 seed, with anisotropy correction performed using anisotropy factors (geometry factor point source approximation). These TPS settings reflect those employed clinically in the Galway Clinic. Figure 5 shows a comparison of sensor measurements with TPS expectation.

### 3D phantom LDR Brachy simulated implant

**Figure 6:**
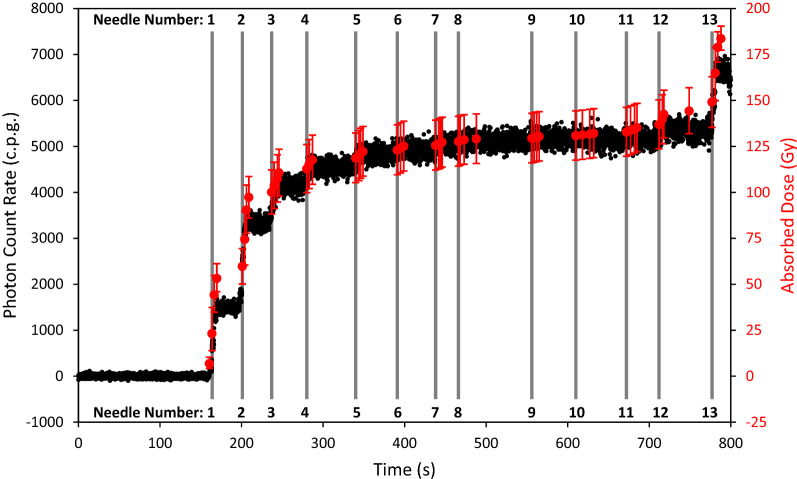
Photon count rate (primary vertical axis) and Absorbed Dose (secondary vertical against) plotted as a function of time, over the course of an I-125 implantation procedure in a 3D anthropomorphic phantom. The real-time PCR data are displayed as black dots, expected absorbed dose readings from the TPS are displayed as solid red circles (one per implanted seed), and vertical grey lines represent each of the 13 peripheral needles.

Accumulated dose to the sensor structure, calculated in the VariSeed TPS, and the real-time PCR data from the sensor, as a function of time are presented in Fig. 6. Accumulated dose values represent the mean dose to the sensor structure in VariSeed, per implanted seed, with “error bars” in this case simply illustrating the minimum and maximum dose values within said structure. The minimum and maximum dose values are displayed in this way to give the reader a representation of the steep dose gradients involved in brachytherapy dosimetry and to illustrate the effect that positional uncertainty can have on measured photon counts.

## Discussion

During the repeatability study, repeated measurements required the sensor to be removed from the phantom prior to each individual measurement, introducing an element of variability due to the repositioning of the sensor on re-insertion within the phantom, relative to the I-125 seed. However, results obtained in this study demonstrate that the output response of the sensor, pre- and post- sterilisation are within the acceptable measurement uncertainty ranging from a maximum standard deviation of 4.7% pre and 5.5% post respectively, indicating that the low temperature sterilisation process does not damage the sensor or reduce performance. Furthermore, analysis of sensor measurements obtained at four angles with respect to the radiation source agree within measurement uncertainty, indicating sensor response uniformity.

Figure 5 shows that the rate of change of the PCR, as a function of distance, is well described by the TG-43 formulism, via the VariSeed TPS, within measurement uncertainty. Within the range of distances considered in this work, the PCR fall-off is dominated by the inverse square law. Furthermore, Fig. 5 illustrates the impact of positional uncertainty on the accuracy of the acquired output measurement, particularly as distance decreases, due to the steep dose gradient. This represents a key challenge when it comes to accurately measuring the dose distribution close to brachytherapy sources. Future work and further development of the optical fibre based system and measurement processes will aim to continue to reduce this positional uncertainty, so as to address this challenge. For example, for the purposes of optical fibre sensor characterisation, replacing the solid PMMA phantom with a watertank would allow for more precise positioning of the sensor with respect to the radiation source. Furthermore, when considering the ultimate goal of transferring this technology to the clinical setting for in vivo patient measurements, it is envisaged that an external tracking system could be implemented, which would allow for precise localisation of implanted sensors within the patient.

From Fig. 6, it is clear that the output signal from the sensor and the expected dose calculated by the TPS initially rise quickly (Needles 1–3), followed by a period of relatively slow increase in PCR/absorbed dose (Needles 4–12), before finally showing a sharp rise again for the final peripheral needle (Needle 13). This behaviour can be explained when one considers the position of the radiation sources (I-125 seeds) with respect to the position of the sensor. As shown in Fig. 5, the PCR falls off quickly with distance.

What can also be seen in Fig. 6 is that in the regions of the steepest dose gradients, the measured PCR seems to under-estimate the expected accumulated dose to the sensor position. It is hypothesised that the observed disagreement between real-time PCR and accumulated dose in these regions is likely due to the angular dependence of the sensor along the longitudinal plane (i.e. the sensor geometry is cylindrically symmetrical so changes in response along the longitudinal plane can be expected) and/or anisotropy in the dose distribution along the longitudinal plane. Future work will consider further characterisation of angular dependence for both polar and azimuthal angles.

For the purposes of this work, however, the objective was to identify if the sensor was capable of accurately identifying when radioactive I-125 sources were introduced into each peripheral needle. When the needles are close to the sensor (≤ approximately 20 mm) it is clear from Fig. 6 that this objective is fulfilled; where sharp rises in PCR are observed at points which correlate with timestamps for the implantation of seeds in a new needle (Needles 1–3 and Needle 13). Where needles are further away from the sensor however, relative increases in the PCR are much smaller, making it difficult to discern the implantation of seeds through a new needle (needles 4–12). This result suggests that future work may consider the implantation of multiple sensors to overcome this limitation. The authors suggest using multiple sensors, as opposed to moving a single sensor for example, since the precision with which the position of the sensor(s) are known is critical to the overall accuracy of the system. Therefore sensors will be positioned and localised at the beginning of a procedure and will remain in position throughout the clinical case, to ensure positional uncertainties are minimised.

Future work will consider characterisation of any energy dependence for the sensor employed in this study and its influence on the conversion process from PCR (c.p.g.) to dose rate (cGy h^−1^). It is worth noting however that previous Monte Carlo modelling work by Meigooni et al^18^ and Weaver et al^19^ have demonstrated that changes in the energy spectra for Iodine 125 are small over the distance range considered, indicating that a correction for energy as a function of distance may not be necessary. The finding presented in this work, is that the fall-off in the PCR as a function of distance agrees with expectation from the TG-43 formulism, seems to be in agreement with this indication.

## Conclusion

Results obtained in this study demonstrate that the output response of the sensor, pre- and post- sterilisation is within the acceptable measurement uncertainty, indicating that the sterilisation process does not damage the sensor or reduce performance. A real time intraoperative LDR prostate brachytherapy treatment in a simulated prostate, using a novel 3D printed anthropomorphic phantom, was performed. Optical fibre measurements demonstrated that the system is capable of accurately identifying when radioactive I-125 sources are introduced into a needle, when the needles are close to the sensor (≤ approximately 20 mm). Future work and areas for further development have also be identified and discussed in this study (e.g. minimising positional uncertainty, further characterisation of angular dependence, and the possible use of a multi-sensor configuration for in vivo measurements, and characterisation of energy dependence).

In the opinion of the authors this study demonstrates the potential of this GOS based optical fibre dosimetry system to be employed as an RTIVD tool, during an LDR prostate brachytherapy implantation procedure. It is minimally invasive, providing additional valuable dosimetry information which will aid the radiation oncologist, ensuring optimum seed placement and good long term clinical outcomes. We believe this system has the potential for further investigation in clinical brachytherapy practice.
